# Exceptionally preserved embryos reveal maternal care in freshwater bivalves since the Cretaceous

**DOI:** 10.1038/s41598-026-56499-1

**Published:** 2026-06-22

**Authors:** Graciela Delvene, Rafael P. Lozano, Martin C. Munt, Aleksandra Skawina

**Affiliations:** 1 Museo Geominero (CN Instituto Geológico y Minero de España, CSIC), Madrid, Spain; 2 School of Environment and Life Sciences, University of Portsmouth, Portsmouth, UK; 3 Faculty of Biology, Institute of Evolutionary Biology, University of Warsaw, Warsaw, Poland

**Keywords:** Fossilized brooding, Evolution, Mesozoic, Unionida, Soft tissue preservation, Ecology, Ecology, Evolution, Zoology

## Abstract

As an adaptive reproductive strategy to their habitat, the majority of freshwater bivalve lineages incubate their larvae in the adults’ gills. The Unionida (pearly mussels) with up to 1000 living species worldwide, are widely accepted as key components of modern freshwater ecosystems. Furthermore, they are unique amongst other freshwater bivalves because their larvae, after finishing maternal incubation, must also parasitize fish to complete their embryonic development and dispersal. Here, we report fossil evidence of a functional freshwater bivalve reproductive system in *Margaritifera valdensis*, a unionoid from the iconic Lower Cretaceous *Iguanodon* locality on the Isle of Wight, southern England. We document four interconnected bioelements of their gill anatomy: gill supports, interlamellar junctions within the gill demibranchs, mineral concretions, and fossilized gill soft tissue. Moreover, diverse developmental stages of brooded embryos and larvae are identified. These data reveal larval incubation within modified gills and evidence a calcium source for their shell formation. These highlight a key evolutionary innovation that facilitated the Mesozoic diversification of unionoid bivalves in calcium-deficient freshwater habitats. Our findings provide the first fossil evidence that by the Early Cretaceous this successful brooding adaptation in freshwater unionoid bivalves developed, providing a significant clue to understanding the evolution and general role of bivalves’ gill anatomy in their function for reproduction.

## Introduction

The reproductive cycle of unionoid bivalves exhibits a strikingly distinctive strategy among invertebrates, closely linked to their successful adaptation to freshwater environments. Larvae initially develop within the adult bivalve gills and subsequently parasitize a host, typically a fish to complete their embryonic development^1^. Our study focuses on the first stage of this cycle: incubation within the adult gills.

The trait of internal offspring incubation is shared with other freshwater bivalves outside the Unionida, including Sphaeridae, Cyrenidae, and some Dreissenidae^2,3^ as well as with a few rare marine bivalves^4,5^. In unionoids, females release oocytes into their gills, where they are fertilized by sperm carried in through filtration from the surrounding water^6^. Maternal care is provided by incubating the developing offspring within specialized brood chambers (marsupia) formed by their modified eulamellibranch gills^7,8^. This condition is shared by all extant Unionida, however, the earliest recognized fossil eulamellibranch gills in the group are from an Upper Cretaceous specimen of *Anodontites freitasi* from Brazil^9^. Earlier reports of phosphatized soft tissue in Triassic bivalves were reported^10^ for related marine genera (Trigoniida). Nevertheless, for unionoids, phosphatized gills of less advanced filibranch anatomy have been described in freshwater *Silesunio parvus* and *Tihkia* sp. from the Upper Triassic of Poland. Such gill anatomy was then likely inherited from their marine ancestors, the Trigoniida^7,11–13^. In both groups, gill supports are basally calcified, which is accepted as a condition that may facilitate fossilization^14^. Eulamellibranch gills differ from filibranch anatomy by the presence of transverse tissue connections (interfilamentary tissue junctions) between gill filaments, while filaments in filibranch gills are connected by interlocking cilia, a consequence of which is easier mechanical disconnection. Although filibranch anatomy does not exclude embryo incubation (however, in the infrabranchial cavity, not inside the gills)^15–16^, eulamellibranch anatomy offers a more suitable framework for the evolutionary development of the modifications required for marsupial formation.

Isolated unionoid larvae (glochidia) have been reported from Tertiary deposits^17^ as well as from Quaternary strata, including the Late Pleistocene and Holocene^18–19^. However, marsupial gills containing developing larvae under maternal care have not been documented to date.

While adults shelter their larvae, they must also contend with the low calcium availability in freshwater habitats; to do so, they produce mineral concretions that serve as calcium reservoirs to supply calcium for larval shell formation^20^. Our work proposes a paradigm shift in the study of fossil bivalves by focusing on mineralized structures other than the shell. Traditionally, only carbonate shells—or occasionally phosphatized tissues—were considered preservable. However, bivalves form additional mineralizations during life^21^ that, under exceptional conditions, can enter the fossil record and be observed with appropriate methodologies. Comparable biomineralizations are also known in extant^22^ and fossil cephalopods^23^.

This study focuses on exceptionally well-preserved margaritiferid *Margaritifera valdensis*^24,25^(Unionida) from the Lower Cretaceous of the Isle of Wight (southern England). This region is renowned not only for its dinosaur fossils, including *Iguanodon*, but also for its diverse non-marine bivalve assemblages and co-occurring fish fossils. Evidence of soft-part preservation in these bivalves was first suggested by W. Bensted and reported by Mantell^26^, who coined the term “Molluskite” for a dark substance composed of approximately 35% “animal carbon”. Martin Whyte and Bruce Runnegar identified gill anatomical features in specimens from the British Museum of Natural History (Natural History Museum, London), but these observations were never formally published^7,20,27–28^.

Our study reinterprets “Molluskite” and analyzes the earliest record of diverse phosphatized soft tissues in these freshwater bivalves. We identify recognizable bioelements of the reproductive system, including interpretable gill marsupia, gonadal tissues associated with mineralized concretions, and distinct embryonic and larval developmental stages. These findings demonstrate that complex maternal care and marsupial brooding in freshwater invertebrates can be directly documented in the Early Cretaceous fossil record, providing new insight into the preservation of reproductive anatomy and redefining the temporal and evolutionary framework of non-marine reproductive strategies.

## Results

**Fig. 1:**
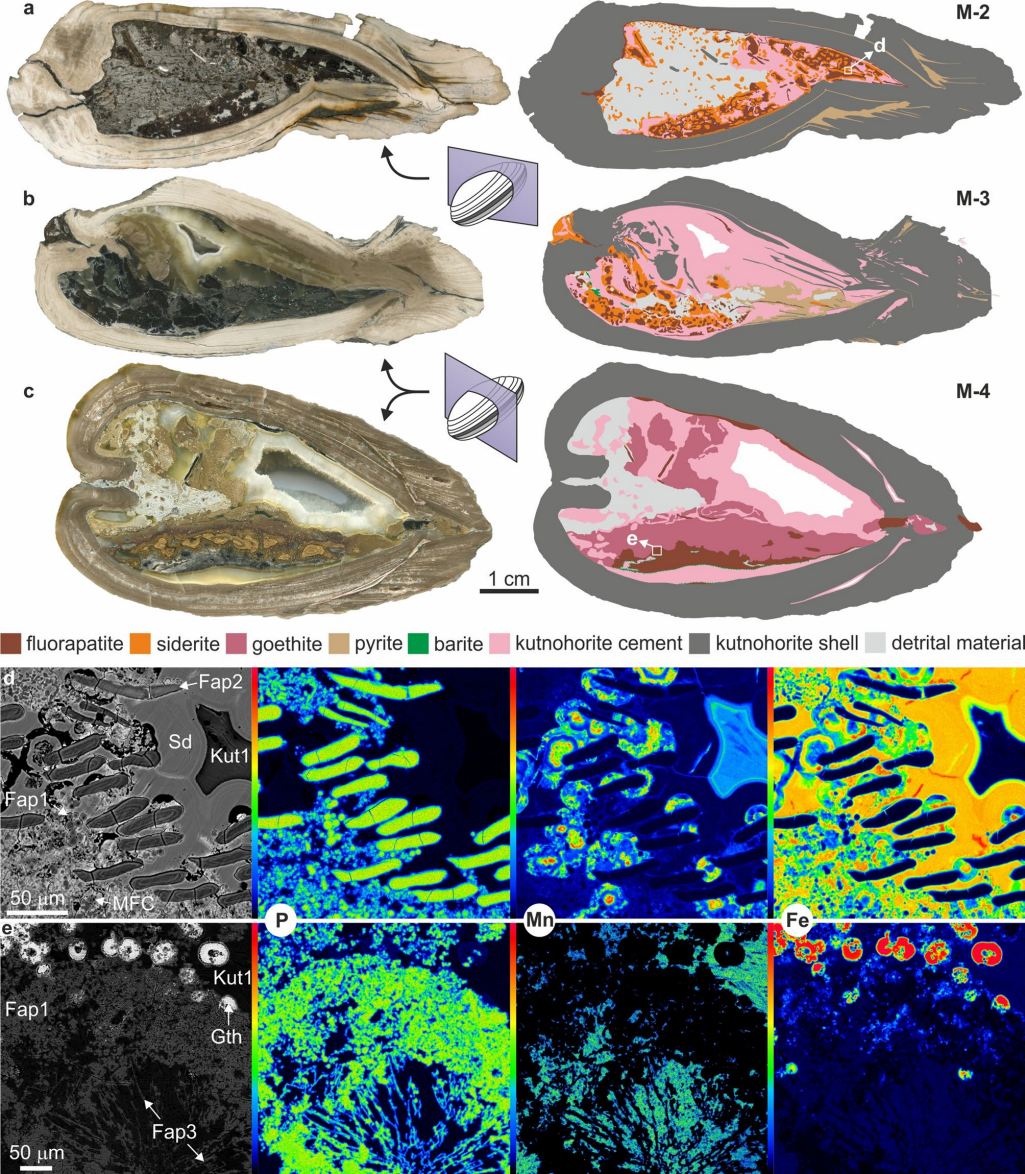
Mineralogy and chemical composition of dorsoventral cross-sections of the fossil bivalves.( **a**, **b**, and **c)**, Cross-sections of specimens M-2, M-3, and M-4, respectively. (**d**, **e**) Backscattered-electron SEM images with compositional maps (P, Mn and Fe) from two selected areas of sections M-2 and M-4, respectively. Fluorapatite: Fap1-4. Fap1: Microspherules (mineral concretions). Fap2: Rods (gill supports). Fap3: Radiating fibrous aggregates (interlamellar junctions). MFC: Mn-Fe-Ca-carbonate (MFC-embryos). Gth: goethite (Gth-embryos). Sd: siderite, Kut: kutnohorite.

Three specimens of *Margaritifera valdensis* underwent dorsoventral cross-sections. For simplicity, shorter field codes are used throughout the text. They show geopetal infills composed of fluorapatite (carbonate-rich variety: francolite), together with siderite or goethite. The porosity is partially filled with detrital material and cemented by kutnohorite with minor pyrite and barite. The original aragonitic shell of the bivalves (Fig. 1a–c) was replaced by kutnohorite, and pyrite grew within the former organic layers^29^. Fluorapatite (Fap) occurs as: microspherules (Fap1), rods (Fap2), radiating fibrous aggregates (Fap3), and massive material surrounding the rods (Fap4). Mn-Fe-Ca-carbonate (MFC) and goethite form pseudospherules (Fig. 1d, e; Table 1).

**Table 1:** Sizes of the bioelements. Measurements are given in µm and refer to the diameter of the sections, except for rods (gill supports), for which values correspond to the thickness of sections taken perpendicular to the longest axis of the rod.

Bioelement	Sample	Max. (µm)	Min. (µm)	Mean (µm)	*n*
Microspherules aggregates (mineral concretions)	M-2 + M-3 + M-4	4	0.5	2.5	1006
Microspherules dispersed (mineral concretions)	M-2 + M-3 + M-4	40	0.5	3.6	1671
Rods (gill supports)	M-2 + M-4	20	8	12.0	102
Radiating fibrous aggregates (interlamellar junctions)	M-2 + M-4	1400	600	1000	32
Radiating fibrous aggregates (interlamellar junctions)	M-3	1000	400	500	36
Polygonal pseudospherules (oogonia)	M-4	15	5	10.0	483
Pseudospherules (incipient embryos)	M-4	15	5	10.8	372
Pseudospherules (mature Gth-embryos)	M-4	30	15	19.2	165
Pseudospherules (mature MFC-embryos)	M-2	45	20	29.3	204
Pseudospherules (larvae)	M-4	100	40	62.4	34

**Fig. 2:**
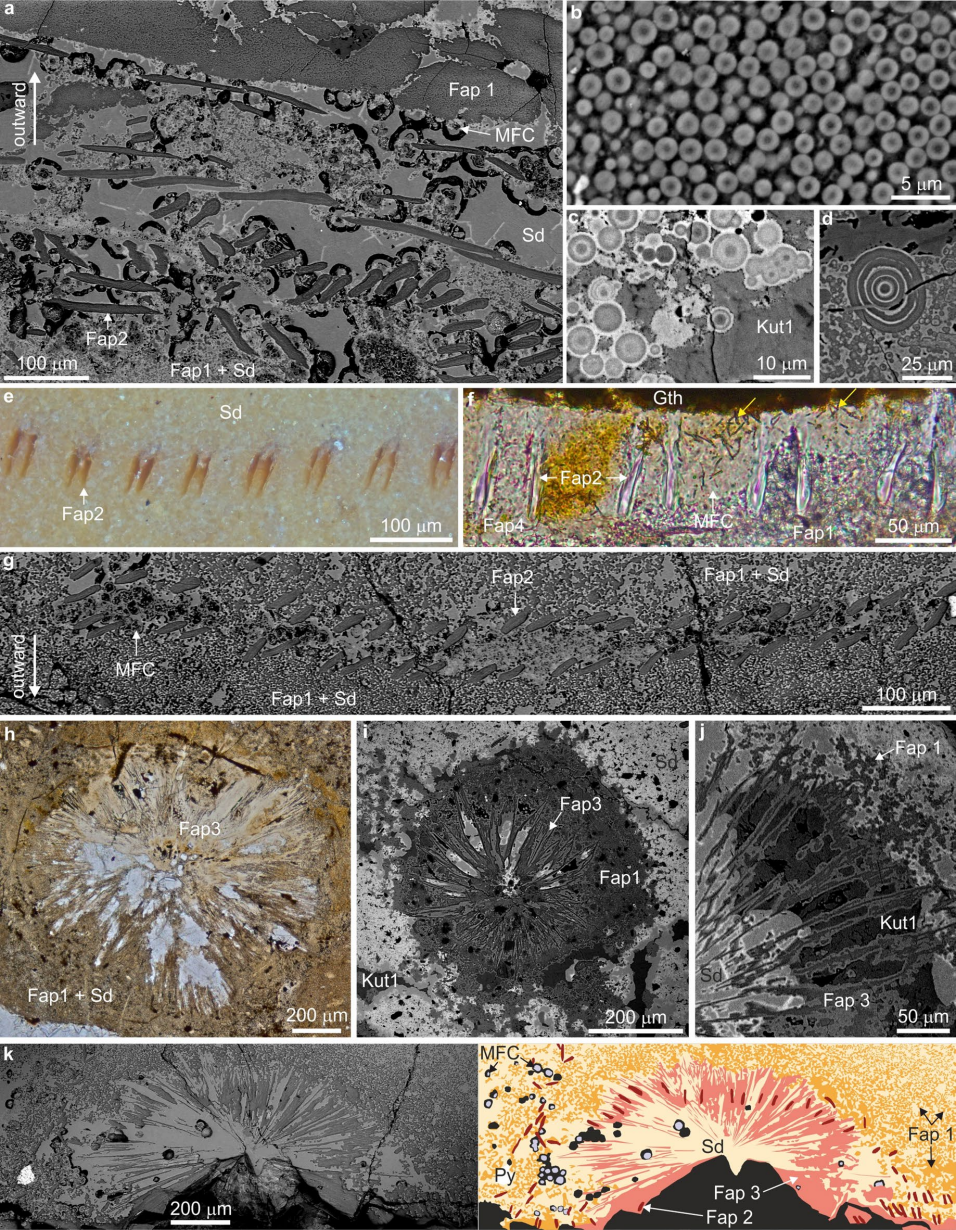
Bioelements preserved in fluorapatite (francolite). (**a**), Microspherules (mineral concretions, Fap1) forming an aggregate toward the bivalve shell and dispersed toward the core in contact with rods (gill supports, Fap2) and Mn-Fe-Ca-carbonate pseudospherules (MFC-embryos). (**b**), Magnified view of the mineral concretions aggregate. (**c**,** d**), Magnified view of the dispersed mineral concretions. (**e**), Simple alignment of gill supports. (**f**), Simple alignment of gill supports in massive fluorapatite (soft tissue, Fap4). The yellow arrows indicate the filaments (filamentous bacteria). (**g**), Double alignment of gill supports with MFC-embryos. (**h**,** i**), Radiating fibrous aggregates (interlamellar junctions, Fap3). (**j**), Detail of the fibres of interlamellar junctions. (**k**), Interlamellar junction with MFC-embryos surrounded by gill supports. Sd: siderite; Kut1: kutnohorite cement. Backscattered-electron SEM images (left) and explanatory diagram of the same images (right). Py: pyrite. (**a**,** b**,** g**,** k**): M-2. (**d**,** e**,** i**,** j**): M-3. (**c**,** f**,** h**): M-4. (**a**,** b**,** c**,** d**,** g**,** i**,** j**,** k**): backscattered-electron SEM images. (**e**): reflected light. (**f**): transmitted light.

Microspherules (Fap1) have a nucleus surrounded by alternating light and dark concentric layers (Fig. 2a–d), and some are composed of two or more individuals (Fig. 2c). In all specimens, they occupy most of the fluorapatite volume, in two distinct sectors with different diameter distributions (Fig. 2a; Table 1). In areas close to the bivalve shell, they form aggregates of uniform size, with all microspherules in mutual contact (Fig. 2b). Toward the interior, they are more widely spaced, show much greater size variability, and are in contact with rods (Fap2) (Fig. 2c). MFC pseudospherules occur within the inner zones of microspherules and rods (Fap1 and Fap2), but also in contact with the outer aggregated zone (Fap3) (Fig. 2a). Because of the section orientations, rods (Fap2) appear in cross-section perpendicular to their long axes, showing regular sizes (Table 1). They align and occur in uniformly spaced pairs, arranged in single (Fig. 2e, f) or double rows (Fig. 2g). Some single alignments are embedded in massive fluorapatite (Fap4) that contains filaments (Fig. 2f). Double alignments contain Mn-Fe-Ca-carbonate (MFC) pseudospherules within them (Fig. 2g). In detail, the rods (Fap2) show concentric zoning, with alternating dark and light layers (Figs. 1d and 2a). Radiating fibrous aggregates (Fap3) are spherical or ellipsoidal. Their diameters are larger in specimens M-2 and M-4 (Fig. 2h) than in specimen M-3 (Fig. 2i) (Table 1). These aggregates may occur within double rod alignments, may contain Mn-Fe-Ca-carbonate pseudospherules (Fig. 2k), and may also be in contact with goethite pseudospherules. The chemical composition of the four types of phosphatic elements includes significant amounts of Fe, S, Mn and Na (Table 2).

**Fig. 3:**
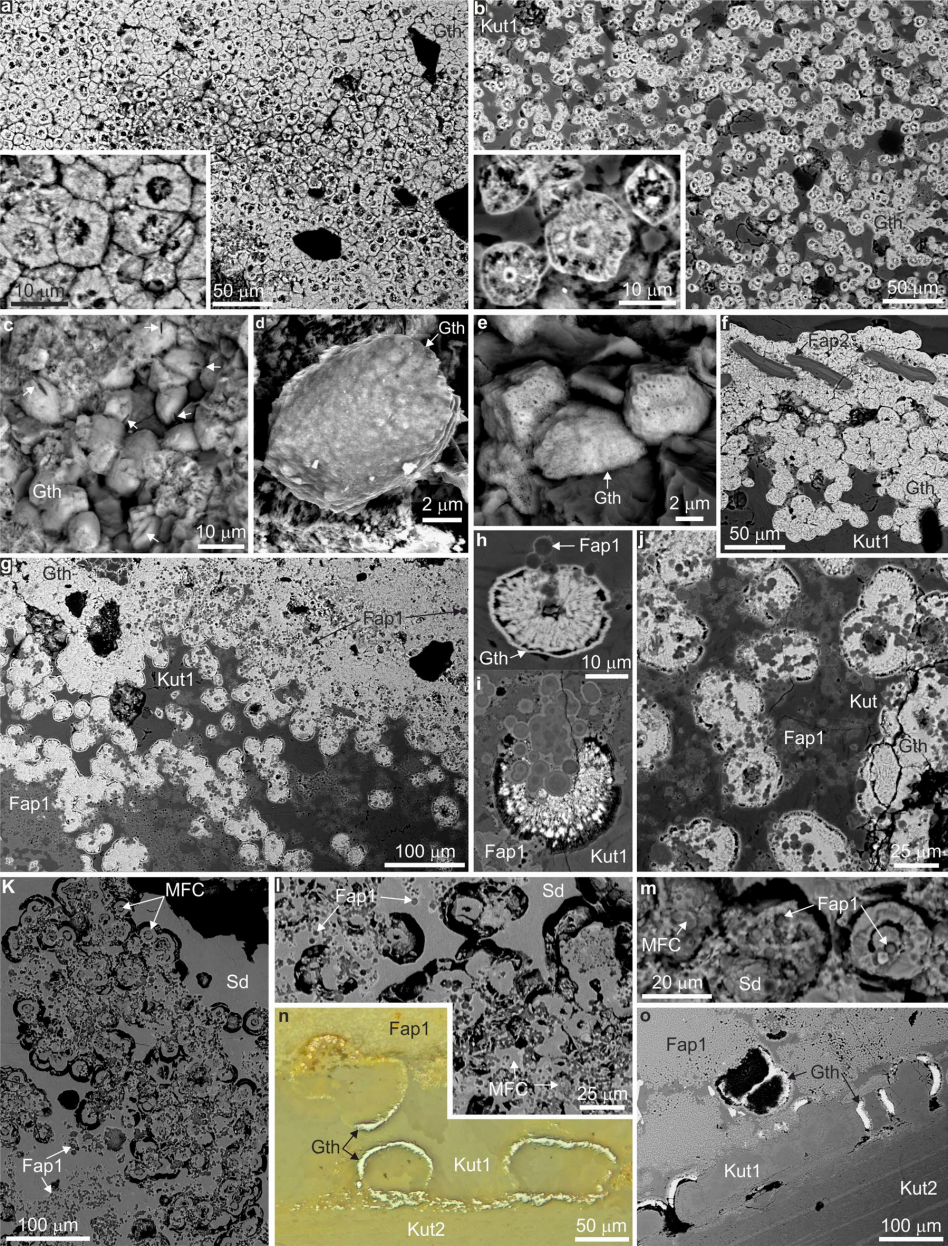
Bioelements preserved in goethite and Mn-Fe-Ca-carbonate. (**a**), Goethite mass composed of polygonal pseudospherules fused together (oogonia in the gonads), with magnified detail. (**b**), Mass of slightly individualized goethite pseudospherules (incipient embryos), with magnified detail. (**c**), Three-dimensional appearance of partially fused incipient embryos. Arrows indicate grooves. (**d**), Individualized incipient embryo with nanoscale protrusions (ornamentation). (**e**), Incipient embryos showing the porosity of the ornamentation. (**f**), Embryos surrounding the rods (gill supports, Fap2). (**g**), Basal boundary of the polygonal mass with microspherules (mineral concretions, Fap1) and larger pseudospherules (Gth-embryos). (**h**,** i**,** j**), Gth-embryos in contact with mineral concretions. (**k**), Group of Mn-Fe-Ca-carbonate pseudospherules (MFC-embryos). (**l**,** m**), MFC-embryos in contact with mineral concretions. (**n**,** o**), Pseudospherules (larvae with open valves; butterfly position). Gth: goethite; Sd: siderite; Kut1: kutnohorite cement; Kut2: kutnohorite shell. MFC: Mn-Fe-Ca-carbonate. M-2: (**k**,** l**,** m**); all others: M-4. (**n**): reflected light; all others: backscattered-electron SEM images.

Pseudospherules are present only in specimens M-2 and M-4, occurring as Mn-Fe-Ca-carbonate in M-2 and as goethite in M-4 (Fig. 1d, e). No pseudospherules were observed in specimen M-3, where only fluorapatite components (Fap1-2-3) are present. The base of the goethite mass (Fig. 1c) is composed of polygonal pseudospherules fused together, with a partially hollow nucleus (Fig. 3a). Most of the goethite volume consists of individualized pseudospherules or clusters of several individuals embedded in kutnohorite. Their sections commonly show two pointed ends and an internal concentric, radiating-fibrous structure (Fig. 3b). The pseudospherules are somewhat faceted and display small grooves (Fig. 3c). At higher magnification, their surfaces show nanometric protrusions (Fig. 3d) with one pore on each protrusion (Fig. 3e). Occasionally, pseudospherules are in contact with aligned rods (Fap2) (Fig. 3f). The basal boundary of the polygonal mass is bordered by larger pseudospherules (Table 1). Some microspherules (Fap1) occur within the polygonal mass, but they are much more abundant at its boundary, where the pseudospherules are larger (Fig. 3g). These pseudospherules also show internal concentric and radiating fibrous structures, with a hollow immediately beneath the outermost concentric layer (Fig. 3h) or on the outermost layer itself (Fig. 3i). They are in contact with microspherules and can enclose them (Fig. 3h–j). Mn-Fe-Ca-carbonate pseudospherules have dimensions similar to those of the goethite pseudospherules (Fig. 3k; Table 1) and exhibit the same textural relationships with the microspherules (Fig. 3l, m). The largest pseudospherules (Table 1) occur detached from the goethite masses, in the upper part of the section (Fig. 1c). They may be single or paired and lie within a narrow space between an aggregate of microspherules and the bivalve shell (Fig. 3n, o). The chemical composition of the goethite includes significant amounts of Al, Si, Mn, Ca, and Mg. The Mn-Fe-Ca-carbonate is an intermediate member of the rhodochrosite-calcite-siderite series, where Mn may be the most abundant cation but is never dominant. It also contains trace amounts of P, Al, Mg, and S (Table 2).

**Table 2:** Mean values obtained by Electron Probe Microanalysis (EPMA) in bivalve sections. Fap1-Fap4: fluorapatite (carbonate fluorapatite, francolite variety). Fap1: microspherules (mineral concretions). Fap2: rods (gill supports). Fap3: radiating fibrous aggregates (interlamellar junctions). Fap4: bulk fluorapatite (fossil soft tissue). Gth (goethite): pseudospherules (oogonium/Gth-embryos/larvae). *For goethite analysis, total Fe is given as Fe₂O₃ to represent Fe³⁺ required by stoichiometry. Sd (siderite): early diagenetic pore-filling cement. MFC (Mn-Fe-Ca-carbonate): pseudospherules (MFC-embryos). Mean of the two analyses with the highest Mn content. Kut1 (kutnohorite): late diagenetic pore-filling cement. Kut2 (kutnohorite): diagenetic shell replacement. Data in wt%. *n* = number of analyses. **CO₃ in fluorapatite was estimated using an EPMA-based stoichiometric approach (A-site cations normalised to 10 atoms per formula unit; CO₃ = 6 − [P + S + Si]; wt% CO₃/CO₂ calculated from formula-unit proportions).

	Fap1	Fap2	Fap3	Fap4	Gth	Sd	MFC	Kut1	Kut2
CaO	51.6	52.1	52.2	52.2	1.1	7.8	16.5	48.7	49.5
MgO	0.1	0.1	0.1	0.0	0.3	0.2	0.2	0.5	0.5
BaO	0.1	0.2	0.1	0.1	0.1	0.1	0.0	0.0	0.1
SrO	0.1	0.2	0.2	0.3	0.1	0.1	0.0	0.1	0.1
SiO_**2**_	0.0	0.0	0.0	0.0	2.5	0.0	0.0	0.0	0.0
Al_**2**_**O**_**3**_	0.1	0.3	0.1	0.1	2.4	0.0	0.2	0.0	0.9
Na_**2**_**O**	0.5	0.5	0.5	0.4	0.0	0.0	0.0	0.0	0.0
FeO	2.0	1.8	1.7	1.7	75.7*	63.1	11.9	1.7	1.5
MnO	0.7	0.8	0.7	0.7	1.5	1.0	26.7	10.3	7.9
P_**2**_**O5**	35.0	33.7	34.3	31.3	0.8	0.6	0.6	0.1	0.0
SO_**3**_	1.4	1.8	1.5	0.9	0.0	0.0	0.1	0.3	0.3
**F**	3.2	3.1	3.1	3.2	0.0	0.1	0.0	0.1	0.1
CO_3_^**^	4.6	5.7	5.4	8.2					
Total	99.3	100.3	99.9	99.2	84.5	73.0	56.3	61.8	61.0
n	1	4	3	1	9	10	2	9	10

## Discussion

Fossil microspherules (Fap1, Fig. 2a–d) are calcium phosphate (fluorapatite) concretions likely produced by bivalves during their life, and are identical to those observed in extant bivalves^20,21^, ^30–35^. Extant and fossil Unionida concretions exhibit similar compositions, including minor elements such as Fe, Mn, S, Na, Mg, Ba and Al^33,35–37^. In both modern and fossil specimens, concretion size and distribution vary by tissue: mantle microspherules are small and clustered, whereas gill microspherules are larger (Table 1), more dispersed, and often include composite forms^20,33,35^.

Fossil aligned rods (Fap2, Fig. 2e–g; Table 1) are mineralized gill supports that demonstrate the presence of eulamellibranch bivalves in the Lower Cretaceous. Extant eulamellibranch gills consist of lamellae joined by connective tissue and supported by uniformly thick chitinous rods, which are partially impregnated with calcium phosphate and contain traces of Fe, Mn, S, Na, Mg and Al^14,33,36,38–40^. In some areas, the tissue attached to the fossil gill supports is preserved together with the filamentous bacteria, likely responsible for its phosphatization^12,41–43^(Fap4, Fig. 2f).

The fluorapatite fibrous radiate aggregates (Fap3) represent fossil interlamellar junctions supporting the two lamellae of each demibranch (Fig. 2h–k) and are interpreted as part of the marsupium to brood the embryos. Although interlamellar junctions in extant Unionida are composed of collagenous connective tissue^40,44^, the presence of calcium phosphate in the fossil fibres (Fig. 2j) suggests that the tissue was likely mineralized in life. As calcium phosphate concretions contact the interlamellar junctions in extant *Margaritifera*, facilitating partial mineralization^33^, this association supports the interpretation of primary fossil mineralization.

**Fig. 4:**
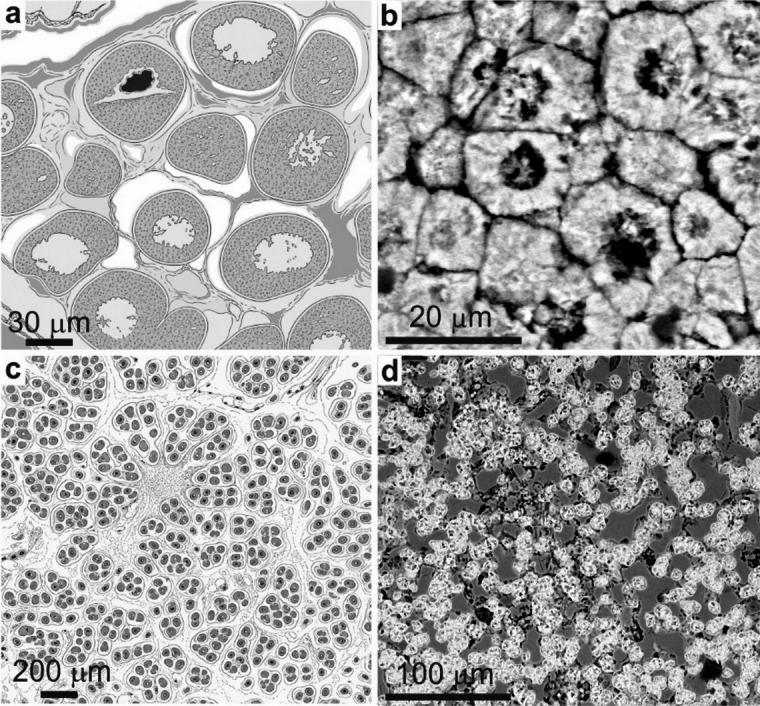
Comparison between early embryonic developmental stages in extant freshwater bivalves and those described in this study. (**a**) detail of the gonads in an extant female *Elliptio complanata*. Modified from^46^. (**b**) polygonal Gth pseudospherules interpreted as oogonia in the gonads of specimen M-4. (**c**) general view of the ovary in an extant female *Margaritifera margaritifera*. Modified from^47^. (**d**) mass of slightly individualised Gth pseudospherules interpreted as incipient embryos in specimen M-4. Panels a and c correspond to AI-assisted interpretative redrawings based on the original micrographs and subsequently modified by the authors.

The pseudospherules represent different ontogenetic stages of a fossil gravid bivalve. In sample M-4, they are preserved in goethite (Fig. 1e) formed through the weathering of siderite^45^. The polygonal mosaic of goethite corresponds to the remains of the female gonad (Fig. 3a), although most oogonia are already individualized as incipient embryos (Fig. 3b). Figure 4 shows a comparison between ovarian sections in extant freshwater bivalves and similar sections examined in this study. Three-dimensional views of the embryos reveal characters that are more evolutionarily advanced than would be expected, including the protoconch apertures (Fig. 3c), external ornamentation, and a visible pore system (Fig. 3d–e).

At the interface with mineral concretions, oogonia enlarge, become rounded, and individualize, representing mature embryos (Gth-embryo) (Fig. 3g). Embryos in sample M-2 (MFC-embryo) exhibit a comparable developmental stage. Their nucleus is composed of Mn-FeCa-carbonate (Fig. 1d) interpreted as the product of bacterially mediated degradation of embryonic soft tissues during early diagenesis^48^. In both cases (Gth–MFC embryos), a hollow layer is present, probably formed by the dissolution of the aragonitic protoconch (Fig. 3h–m).

Gth and MFC-embryos occur in contact with microspherules (Fig. 3g–m), and fossil sections show that these microspherules are present both outside and within the embryos, indicating their incorporation during early development (Fig. 3h, i, m). This pattern is consistent with the strategy observed in extant Unionida, in which mineral concretions provide the calcium required for protoconch formation, initiated in early embryos through aragonite precipitation^20,49–50^. In living Unionida, however, the mechanism by which calcium is transported from microspherules across the vitelline membrane remains unresolved^49^. This fossil record provides the first evidence of the incorporation of mineral concretions from the embryonic stage for shell formation in Unionida bivalves.

**Fig. 5:**
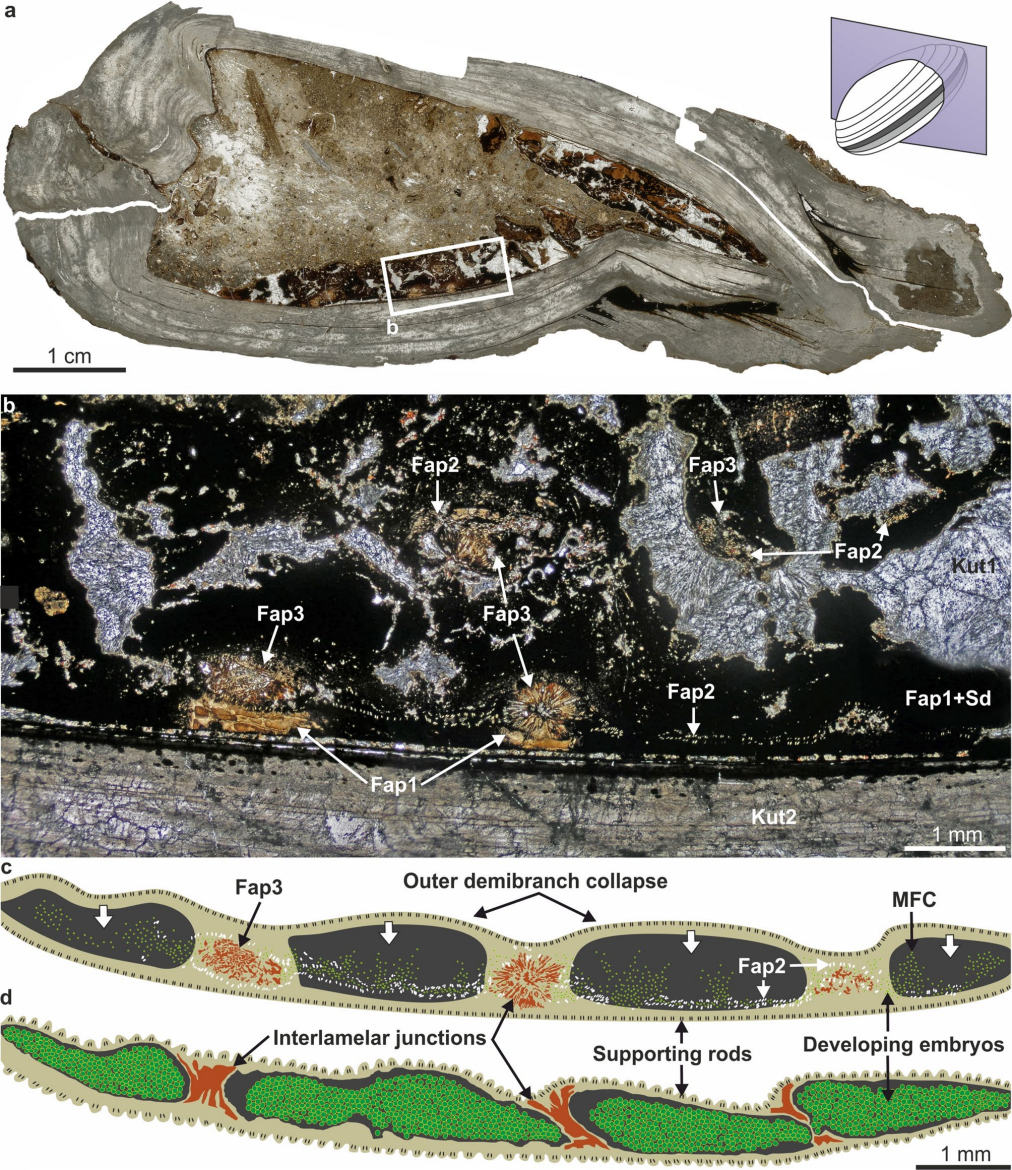
Comparison between a living gravid demibranch and the collapsed demibranch of specimen M-2. (**a**) Thin section of the bivalve. (**b**) Detail of the basal sector showing the interlamellar junctions (Fap3) surrounded by aligned and collapsed branchial supports (Fap2) of the outer demibranch. The interlamellar junctions rest on mantle remnants formed by agglomerated mineral concretions. All elements are enclosed in a matrix of siderite (Sd) + mineral concretions (Fap1). (**c**) Reconstruction of the demibranch prior to collapse. Red areas represent the hypothetical marsupium filled with MFC-embryos, and white arrows indicate the direction of collapse. The positions of MFC-embryos are also shown. (**d**) Outer demibranch of *Margaritifera auricularia* (image modified after Soler et al.^8^).

Gth and MFC-embryos are situated within the gills, in contact with the gill supports (Figs. 2a and g and 3f) and the interlamellar junctions (Figs. 1e and 2k), indicating that they were housed in the marsupium before the reproductive system collapsed following the bivalve’s death (Fig. 5).

A possible inorganic diagenetic origin for the pseudospherules can be ruled out based on several lines of evidence. Firstly, the structures are preserved in two distinct mineral phases in different specimens (MFC in M-2 and Gth in M-4), yet they exhibit comparable sizes and morphologies when associated with the mineral concretions (Figs. 3g–m; Table 1). If these structures resulted from purely inorganic mineral growth, significant differences in size and morphology would be expected between the two mineralisation pathways. Secondly, both Gth and MFC pseudospherules display a hollow outer layer (Figs. 3h, k–m), interpreted as the space left after dissolution of the aragonite protoconch. An exclusively diagenetic origin would more likely produce completely mineral-filled spheroidal bodies. Thirdly, Fap elements occur in all three bivalves, but pseudospherules are absent in specimen M-3, which also shows reduced radiating fibrous aggregates (Table 1). If the pseudospherules had formed through inorganic diagenetic precipitation, similar structures would also be expected in M-3. Finally, the Gth pseudospherules preserve unequivocally biological features, including protoconch apertures (Fig. 3c), external ornamentation with pore systems related to larval shell surfaces (Figs. 3d–e), and butterfly-shaped glochidia morphologies representing articulated valves (Figs. 3n–o), which cannot be explained by inorganic mineral growth alone. Their confinement to marsupial gill regions, in close association with interlamellar junctions and gill supports, further supports a biological rather than diagenetic origin.

Occasionally, more developed glochidia occur in a “butterfly position” between the outer mantle (rich in mineral concretions) and the shell surface (Fig. 3n–o). This discovery represents the earliest and first fossil record of glochidia preserved inside bivalves actively brooding their offspring.

The absence of embryos or larvae in specimen M-3 suggests that it either released them or never produced any. The abundance of dispersed mineral concretions supports the latter, as these structures disappear after reproduction. Another criterion indicating that specimens M-2 and M-4 were brooding, whereas M-3 was not, concerns the interlamellar junctions. In extant marsupial gills, these junctions thicken to support developing embryos, and their comparatively reduced thickness in M-3 relative to M-2 and M-4 (Fig. 2h–k) is consistent with a non-reproductive state,^27,51–52^.

Gill brooding emerges as a fundamental reproductive and survival strategy for freshwater bivalves, representing a key innovation that drove the Mesozoic diversification of unionoids in calcium-deficient freshwater habitats. Crucially, this insight was made possible exclusively through the methodology applied in this study, which revealed fine-scale reproductive structures in fossil specimens. These findings provide new insight into the preservation of reproductive anatomy in freshwater unionoids and their evolutionary implications.

## Materials and methods

Three fossil bivalves were embedded in epoxy resin and subsequently cut along the dorsoventral direction. After cutting, two specimens exhibited very dark brown geopetal fills (M-2 and M-3), whereas the remaining specimen showed a light brown geopetal fill (M-4). From specimen M-2, we prepared a large-format thin section encompassing the complete shell section  (10 × 4 cm). From specimens M-3 and M-4, we prepared one standard-size thin sections  (4.8 × 2.8 cm) from each specimen, selecting areas of interest within the geopetal fills. In addition, we prepared one thick section (~ 5 mm in thickness) parallel to the initial cut from specimen M-2, and three similar sections from specimen M-4. For specimen M-3, one comparable section was obtained, as well as an additional section of the same thickness but oriented perpendicular to the initial cut. All preparations were polished using progressively finer diamond abrasives under water cooling, followed by final polishing with aluminium oxide on felt. Specimens M-2 (MGM 7958X), M-3 (IWCMS:2025.160), and M-4 (IWCMS:2025.161) are curated in the Museo Geominero (IGME, CSIC, Madrid, Spain), abbreviated MGM, and the Dinosaur Isle Museum (Sandown, Isle of Wight, United Kingdom), abbreviated IWCMS. 

Samples were examined using Leica DMLP and Olympus BX51 optical microscopes (Museo Geominero, IGME–CSIC, Madrid, Spain) and a Keyence VHX-7000 digital microscope (Institute of Evolutionary Biology, Faculty of Biology, University of Warsaw, Warsaw, Poland), under transmitted and reflected light. Polished sections from specimens M-2, M-3 and M-4, and freshly broken samples from specimen M-4, all coated with graphite, were analysed using a JSM-6010 PLUS/LA scanning electron microscope equipped with an energy-dispersive X-ray spectroscopy (EDS) microanalyzer, a backscattered electron detector (BSE), and a secondary electron (SE) detector at CN-IGME, CSIC facilities (Tres Cantos, Madrid, Spain). To obtain nanometre-scale morphological details, selected freshly broken samples were additionally examined using a JEOL JSM-IT700HR scanning electron microscope at the National Centre for Electron Microscopy, Complutense University of Madrid (Spain).

The different minerals composing the geopetal fills and the bivalve shells of all samples were analysed by X-ray diffraction (XRD) and electron microprobe analysis (EMP). XRD analyses were performed using a X’Pert PRO diffractometer (Panalytical) with a copper tube, a graphite monochromator and automatic divergence slit at the Centro Nacional Instituto Geológico y Minero de España, Consejo Superior de Investigaciones Científicas (CN IGME, CSIC, Tres Cantos, Madrid) facilities. Identifications were made using X’Pert High Score software by Panalytical and the PDF-2 (ICDD) database. EMP measurements were performed using a JEOL JXA-IHP200F electron microprobe operated at 15 kV and 20 nA, with a beam diameter of 1-5 μm, at the National Centre for Electron Microscopy, Complutense University of Madrid (Spain). Elemental detection limits were calculated from background count statistics and converted to oxide equivalents using stoichiometric factors. Iron is reported as FeO and phosphorus as P₂O₅. Detection limits were approximately as follows: 140 ppm for CaO, 149 ppm for MgO, 726 ppm for BaO, 840 ppm for SrO, 428 ppm for SiO₂, 246 ppm for Al₂O₃, 135 ppm for Na₂O, 1286 ppm for FeO, 349 ppm for MnO, 344 ppm for P₂O₅, 300 ppm for SO₃ and 310 ppm for F.

During the preparation of this manuscript, generative artificial intelligence tools, specifically ChatGPT (GPT-5, OpenAI), were used to enhance the clarity and overall readability of the text. The tool provided suggestions for grammatical corrections, as well as improvements in word choice and sentence structure for a limited number of selected sentences in order to better communicate our findings. In addition, ChatGPT was used to assist in the preparation of the interpretative redrawings shown in panels a and c (Fig. 4) which were based on the original micrographs and subsequently reviewed and modified by the authors. The authors retain full responsibility for the content and conclusions presented in this manuscript.

## Data Availability

The datasets used and/or analysed during the current study available from the corresponding author on reasonable request.
